# Analysis Method of Bending Effect on Transmission Characteristics of Ultra-Low-Profile Rectangular Microstrip Antenna

**DOI:** 10.3390/s22020602

**Published:** 2022-01-13

**Authors:** Jiaying Zhang, Jin Huang, Peng Sun, Fanbo Meng, Jie Zhang, Pengbing Zhao

**Affiliations:** Key Laboratory of Electronic Equipment Structure Design, School of Mechanical and Electrical Engineering, Xidian University, Ministry of Education, Xi’an 710071, China; 19041110403@stu.xidian.edu.cn (J.Z.); psun_1@stu.xidian.edu.cn (P.S.); fbmeng@xidian.edu.cn (F.M.); jiezhang1987@xidian.edu.cn (J.Z.); pbzhao@xidian.edu.cn (P.Z.)

**Keywords:** cylindrical-rectangular cavity, effective dielectric constant, equivalent circuit model, ultra-low-profile microstrip antenna

## Abstract

With the advent of wearable communication devices, microstrip antennas have developed multiple applications due to their ultra-low-profile properties. Therefore, it is essential to analyze the problem of frequency shift and impedance mismatch when the antenna is bent. For the case of a rectangular patch antenna E-plane bent on the cylindrical surface, (1) this paper introduces the effective dielectric constant into the cavity model, which can accurately predict the resonance frequency of the antenna, and (2) according to the equivalent circuit model of the antenna resonance mode, the lumped element parameters are calculated based on the above effective dielectric constant, so that impedance characteristics and the S-parameter matching the port can be quickly constructed. From the perspective of circuit frequency characteristics, it explains the change in the transmission performance of the curved antenna. The experimental results show that the maximum difference between the experimental and theoretical calculation frequencies is less than 1%. These results verify the validity and applicability of the theory in the analysis of ultra-low-profile patch antennas and wearable electronic communication devices. It provides a theoretical basis for the fast impedance matching of patch antennas under different working conditions.

## 1. Introduction

With the development of various applications such as wireless body area networks, the antenna technology in wearable devices is becoming more and more compact, flexible, and low-profile [1]. The flexible dielectric substrate (such as film polymers and textiles) microstrip antenna is an ideal choice for wearable antennas [2,3]. On curved surfaces of the human body, the film substrate antenna not only satisfies the above requirements, but also has the advantages of easy integration with other microwave modules [4,5], low cost, and easy installation, design, and manufacturing. However, in the course of use, the reliability of the film antenna has increasingly become a serious issue [6]. Due to specific working conditions, a wearable film antenna will inevitably bend during usage; thus, its expected transmission performance will significantly degrade compared with the same antenna in a flat state, or even fail to function normally. Further, the maximum gain of the antenna will be less affected by the wearing environment. Even if the antenna is designed to allow for a certain bending radius, the bending angle changes owing to human activities. Therefore, it is essential to analyze the frequency offset phenomenon and its influence on the transmission performance caused by the antenna bending [7].

For the design and transmission characteristics of curved antennas, the current mainstream analysis method is to use the High-Frequency Structure Simulator (HFSS) for full-wave simulation [8,9,10,11]. However, for film antennas, the finite element method cannot realize the mechanism analysis between antenna bending and performance changes, and the ultra-thin characteristics force the finite element mesh to be extremely fine, resulting in a long simulation time. These have led to the lack of rules and unbearable waiting time in the structural optimization design of the conformal antenna. Previous research was dedicated to providing the influence of bending on the transmission characteristics. In [12,13], a cylindrical-rectangular cavity model of a curved patch antenna was proposed, but the frequency deviation of the curved antenna was not found and no experiment was provided. In [14], the effective dielectric constant was introduced to analyze the impedance characteristics of the microstrip line, but the electrical performance of the antenna as a load was not analyzed. The authors of [15] introduced the concept of the bending angle of the microstrip antenna and used the equivalent circuit model for analysis. However, the mathematical relationship between bending angle and frequency has not been determined, and it is not possible to quickly construct S-parameters.

This study systematically and comprehensively analyzes the bending effect on the transmission performance of an ultra-low-profile rectangular microstrip antenna. The remainder of this paper is organized as follows. In Section 2, the boundary conditions of the model are described, and the effective dielectric constant is introduced; then, the mathematical relationship between the bending angle and the predicted frequency is presented. In Section 3, the lumped element parameters of the equivalent circuit model are calculated from the effective dielectric constant. Impedance characteristics and the S-parameters that match the port are briefly constructed. In Section 4, supporting molds and film antenna prototypes are fabricated, and the data are recorded using a vector network analyzer. Section 5 presents the conclusion of the study.

## 2. Patch Antenna Bending Model

### 2.1. Effective Dielectric Constant Frequency Prediction Model

We assume that during the bending process, the stretch on patch and compression of the substrate are negligible, and the dielectric properties of the material remain unchanged. The curved antenna is geometrically modeled as a cylindrical-rectangular cavity. The eigenvalue, *k*, and the effective dielectric constant, $\varepsilon_{eff}$, are obtained by solving the vector potential using the Helmholtz equation corresponding to different bending angles. The eigenfrequency of the bending cavity is predicted by an analytical formula.

**Figure 1:**
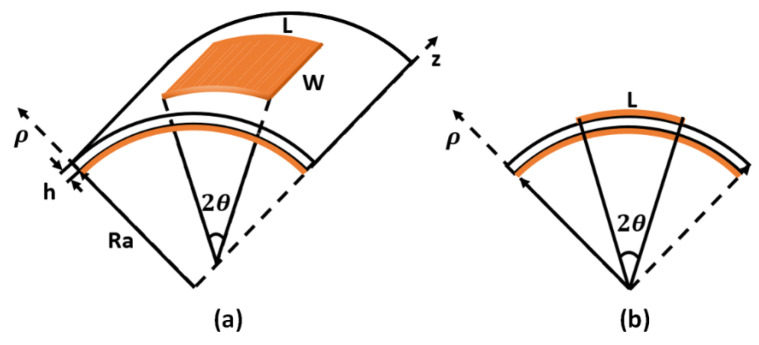
On the cylindrical surface, a rectangular patch antenna is curved along the E-plane: (**a**) geometric model and (**b**) main view.

The cavity geometrical model of the patch antenna can be visualized in a cylindrical coordinate system, as shown in Figure 1. The cylindrical surface to which the rectangular patch is attached takes the z-direction as the axis, *L* is the resonance length *λ*/2, *W* is the width of the antenna, *h* is the thickness of the substrate, Ra is the radius of the cylindrical surface, and the antenna bending angle: 2*θ* = *L*/(*Ra* + *h*).

Inside the cavity, the electric vector potential, $\vec{F}$, and magnetic vector potential, $\vec{A}$, satisfy the Helmholtz equation:

(1) $$\nabla^{2}\vec{F}+k^{2}\vec{F}=0,\nabla^{2}\vec{A}+k^{2}\vec{A}=0$$

The cylindrical coordinate representation of the TM mode equation satisfying the magnetic boundary condition is:

(2) $$\vec{F}=0,\vec{A}=\hat{z}\psi$$

(3) $$\frac{1}{\rho }\frac{\partial }{\partial \rho }\left(\rho \frac{\partial \psi }{\partial \rho }\right)+\frac{1}{\rho^{2}}\frac{\partial^{2}\psi }{\partial \varphi^{2}}+\frac{\partial^{2}\psi }{\partial z^{2}}+k^{2}\psi =0$$

The variable separation method [16] was used to simplify the equation. We ignore the fringe electric field of the microstrip patch, and the electric field around the patch will be perpendicular to the patch surface and the ground plane. The boundary of the cylindrical-rectangular cavity includes the patch and the ground plane (ρ=Ra, ρ=Ra+h), which satisfy the perfect electrical conductor (PEC) boundary conditions, and the four walls between the conductors (ϕ=0, ϕ=2θ, z=0, z=W), which satisfy the perfect magnetic conductor (PMC) boundary conditions [12]. Then, the boundary conditions of the electric potential field in the TM mode can be expressed as:

(4) $$\psi {|}_{\rho =Ra}=0\;R\left(a\right)=A_{v}J_{v}\left(kRa\right)+B_{v}N_{v}\left(kRa\right)=0$$

(5) $$\psi {|}_{\rho =Ra+h}=0\;R\left(b\right)=A_{v}J_{v}\left(k\left(Ra+h\right)\right)+B_{v}N_{v}\left(k\left(Ra+h\right)\right)=0$$

Here, $J_{v}$ and $N_{v}$ are the Bessel functions of the first and second types, respectively, where ν=mπ/2θ is the order of the Bessel function, and the eigenvalues, k, satisfy the condition $J_{v}\left(k\left(Ra+h\right)\right)N_{v}\left(kRa\right)-J_{v}\left(kRa\right)N_{v}\left(k\left(Ra+h\right)\right)=0$. Since the field on the cavity surface, S, either satisfies the short-circuit boundary condition (n×E=0), or satisfies the open-circuit boundary condition (n×H=0), the eigenvalue, k, of the Helmholtz equation can also be expressed as:

(6) $$k^{2}=\frac{\int_{V}^{\;}{\left|\nabla \times E\right|}^{2}dV}{\int_{V}^{\;}E^{2}dV}$$

The eigenvalue, *k*, represents the wave number per unit length when the wave propagates in the medium, k=2π/λ. The wave number $k_{0}$ in a vacuum is $2\pi /\lambda_{0}$. For the cavity model under the boundary conditions of different bending angles, the change in eigenvalue, k, reveals a change in the propagation wavelength, λ, in the medium. Since electromagnetic waves of the same frequency have the same phase velocity: $\nu =\lambda f=1/\sqrt{\mu \varepsilon }$, we introduce the effective dielectric constant [14]:

(7) $$\varepsilon_{eff}={\left(k/k_{0}\right)}^{2}$$

The resonant frequencies of different radiation modes, TM*_mnp_*, calculated by the plane cavity model are:

(8) $$f=\frac{1}{2\pi \sqrt{\mu_{0}\varepsilon_{0}\varepsilon_{r}}}\cdot \sqrt{{\left(\frac{m\pi }{L}\right)}^{2}+{\left(\frac{n\pi }{W}\right)}^{2}+{\left(\frac{p\pi }{h}\right)}^{2}}$$

According to the effective dielectric constant, $\varepsilon_{eff}$, obtained by Equation (7), the above equation can be transformed into:

(9) $$f=\frac{1}{2\pi \sqrt{\mu_{0}\varepsilon_{0}\varepsilon_{eff}}}\cdot k_{plane}$$

When the flat antenna works in TM_10_ mode, $k_{plane}=\sqrt{{\left(\frac{m\pi }{L}\right)}^{2}+{\left(\frac{n\pi }{W}\right)}^{2}+{\left(\frac{p\pi }{h}\right)}^{2}}=\pi /L$.

### 2.2. Numerical Results and Comparison

According to [15] and the simulation results, two conclusions are drawn: (1) E-plane bending more significantly influences the frequency of patch antennas than H-plane bending, and (2) when the E-plane bending angle 2θ ranges from 0° to 35°, the excited mode remains in the basic mode, otherwise a higher mode is excited. Therefore, this article exemplifies the phenomenon of frequency deviation and transmission characteristics change of the patch antenna curved along the E-plane on the cylindrical surface with a bending angle of 0°–35°. Larger bending angles will excite higher modes, such as the TM_11_ mode. Similar to the fundamental mode, by changing the integers m and n in Equation (9), the resonance frequency of the higher-order mode can be predicted.

**Figure 2:**
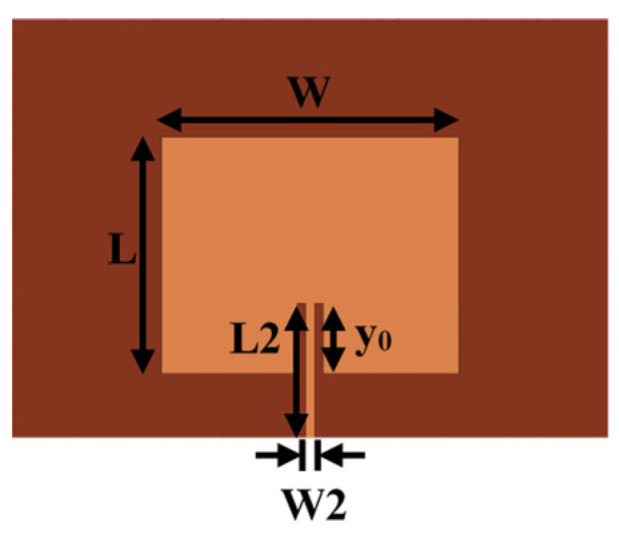
Antenna design parameters based on PI substrate.

**Figure 3:**
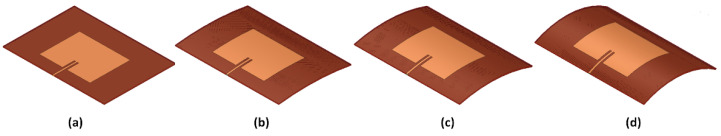
Antenna curved on the cylindrical surface with different E-plane bending angles: (**a**) 0°, (**b**) 10°, (**c**) 20°, and (**d**) 35°.

As shown in Figure 2, a microstrip rectangular patch antenna with an operating frequency of 5.8 GHz was designed. The patch size L=13.5 mm, W=17 mm, the microstrip line size L2=7.7 mm, W2=0.5 mm, and the distance, y_0_, from the feed point to the edge of the patch is 4 mm. A polyimide (PI) film with a thickness of 0.254 mm was used as the substrate, where the size is 27 mm × 34 mm, and the dielectric constant is 3.6. As shown in Figure 3, we assumed that the elastic deformation of the model was negligible. A full-wave simulation model of the antenna with different bending angles on the cylindrical surface was created using HFSS.

**Figure 4:**
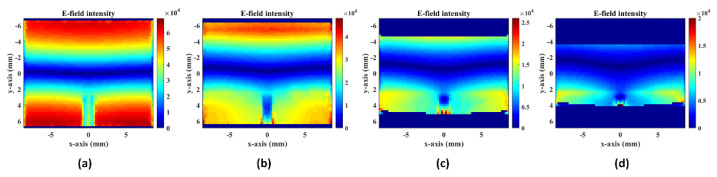
Electric field intensity of the center diagram in ρ-direction with different bending angles: (**a**) 0°, (**b**) 10°, (**c**) 20°, and (**d**) 35°.

A simplified bending cavity model was established in the HFSS eigenmode solver for the microstrip patch, and the boundary conditions (4) and (5) are satisfied at the same time to obtain the electric field, E. With different E-plane bending angles, Figure 4 selects the center diagrams of the model in the ρ-direction, showing the solution results of the electric field intensity. 

**Figure 5:**
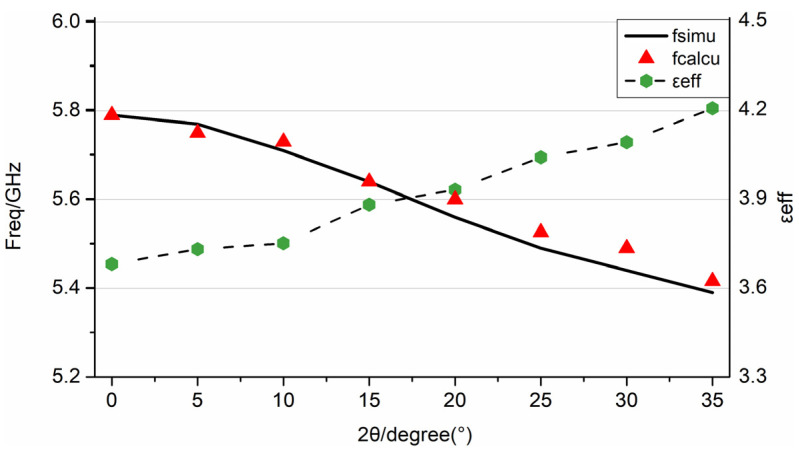
Comparison of full-wave simulation frequencies and the theoretically predicted frequencies with different bending angles, represented by the solid black line and red marks, respectively.

The resonant frequencies under different bending conditions were extracted from the full-wave simulation results. As shown in Figure 5, the resonant frequency shifts to a lower value within the range of the bending angle from 0° (flat) to 35° (slightly bent). The resonant frequency of different bending angles ($f_{2\theta }$) is normalized to the original resonant frequency in the flat condition ($f_{plane}$), expressed as $f_{2\theta }/f_{plane}$, and the 2θ=35° bending angle generates up to −6.91% of the normalized frequency shift. This phenomenon can be explained by the fact that E-plane bending affects the current path, especially for the fundamental mode of resonance. Simultaneously, the curved patch also changes the fringe field of the radiating edge, thereby affecting the effective dielectric constant of the antenna [17].

For the small bending condition (2θ=0°−35°), the antenna was in TM_10_ mode. As the bending angle increased, the eigenvalue and the effective dielectric constant gradually increased, while the resonance frequency gradually decreased. As shown in Figure 5, the frequencies predicted by the effective dielectric constant method and the full-wave simulation results were very consistent, with a maximum error of 0.92%.

## 3. Equivalent Circuit Model

In this section, the generalized equivalent circuit model is introduced to explain the effect of bending on the patch antenna transmission characteristics. The effective dielectric constant, $\varepsilon_{eff}$, under different bending angle conditions was used to calculate parameters of lumped elements, and the circuit was matched to a single-port network with a terminal impedance of 50 Ω. According to the different parameters of lumped elements, the resonant frequency of the circuit can predict the antenna resonant frequency and construct the frequency-related impedance characteristics and S-parameters. 

**Figure 6:**
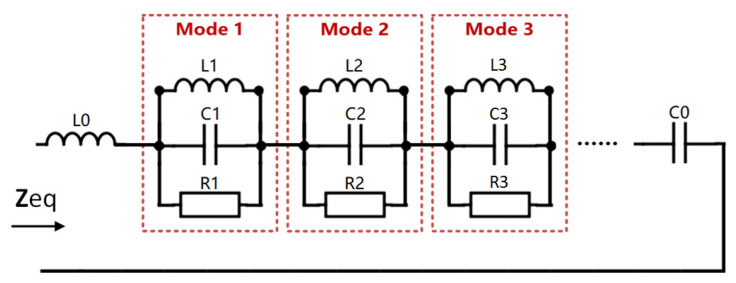
Equivalent circuit model of a single resonant mode antenna element.

As shown in Figure 6, this equivalent circuit topology model has been proven to help analyze microstrip antennas, where *L*_0_ and *C*_0_, respectively, represent the inserted inductance and capacitance that conform to the impedance behavior of the feed point [18]. Under small bending conditions, the antenna is in the basic resonant mode. The influence of higher-order resonant modes can be ignored because of the orthogonality between the resonant modes. Each resonance mode was modeled as an RLC parallel resonance circuit. Here, the capacitance value, $C_{calcu}$, is determined by the effective dielectric constant, $\varepsilon_{eff}$, and the flat capacitor formula, and the inductance value, $L_{calcu}$, is calculated by the flat resonant frequency, $f_{plane}$, and the flat capacitance value, $C_{plane}$. In this example, $L_{calcu}=L_{plane}=0.0303nH$, and the resistance, R, is calculated based on the Q value [19]. The general expression of the equivalent circuit input impedance and return loss is:

(10) $$Z_{eq}=j2\pi fL_{0}+\frac{1}{j2\pi fC_{0}}+{\sum_{i=1}^{n}\left(\frac{1}{R_{i}}+\frac{1}{j2\pi fL_{i}}+j2\pi fC_{i}\right)}^{-1}$$

(11) $$Z_{eq}=\sqrt{R^{2}+X^{2}}\;\Gamma =\frac{Z_{eq}-Z_{0}}{Z_{eq}+Z_{0}}\;RL(\mathrm{dB})=-20\mathrm{lg}|\Gamma |$$

**Figure 7:**
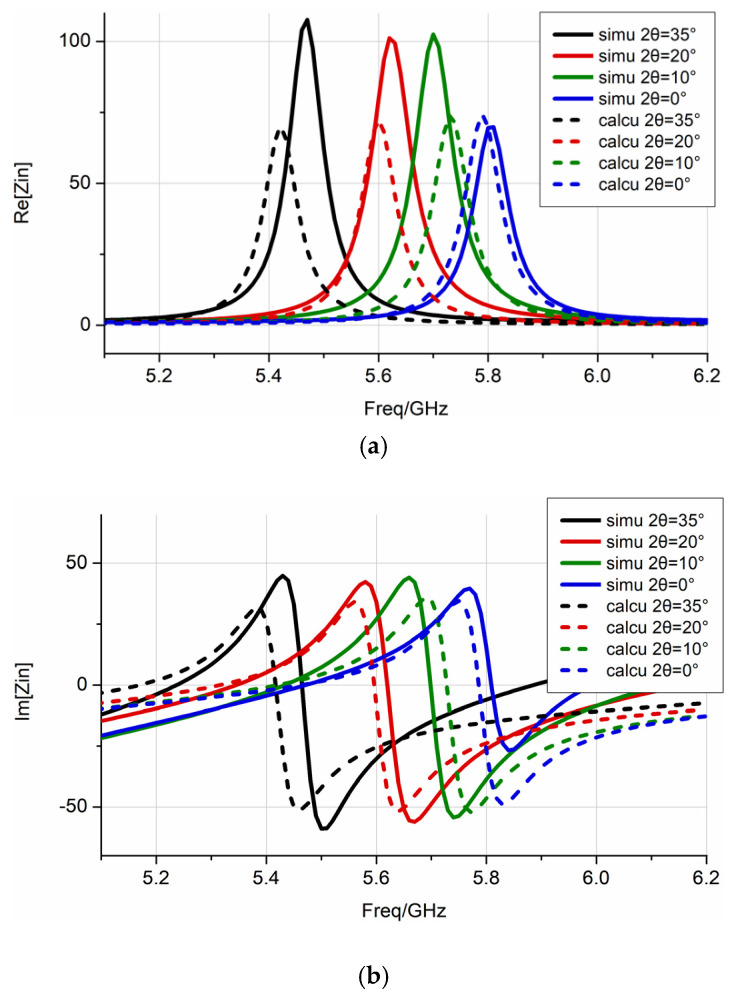
The impedance characteristics under different bending angles by full-wave simulation and equivalent circuit constructed are represented by solid and dashed lines, respectively: (**a**) real part; (**b**) imaginary part.

**Figure 8:**
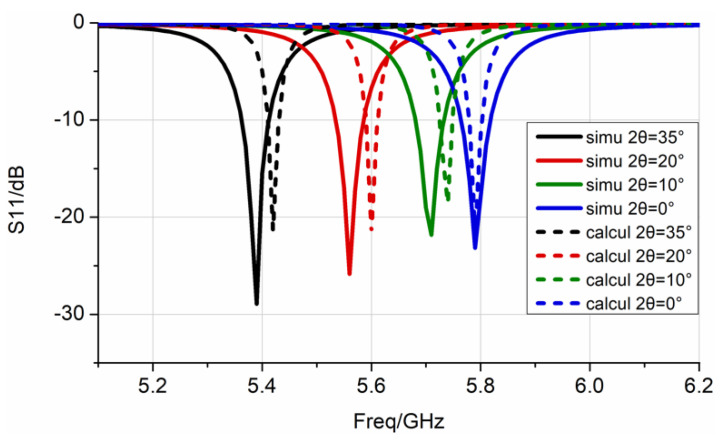
The S-parameters under different bending angles by full-wave simulation and equivalent circuit constructed are represented by solid and dashed lines, respectively.

Table 1 lists the calculation results of the effective dielectric constant, $\varepsilon_{eff}$, which were obtained by Equation (7), and the parameters of lumped elements under different E-plane bending angles. The results reveal that as the bending angle and the effective dielectric constant, $\varepsilon_{eff}$, increased, the equivalent capacitance of the antenna element increased, while the equivalent resistance decreased. As shown in Figure 7, compared with the impedance characteristics obtained by the full-wave simulation sweep frequency process, the impedance characteristics of the equivalent circuit constructed according to Equation (10) are basically the same. This shows that using the effective dielectric constant method to determine the parameters of lumped elements can reflect the impedance characteristics of the antenna. As shown in Figure 8, according to the actual needs of the project, the equivalent circuit was matched with a 50 Ω port. Equation (11) calculates the S-parameter based on the equivalent circuit model. It can be seen that the lowest point of the constructed S-parameter is basically consistent with the resonant frequency of the full-wave simulation, and the difference in amplitude is related to the setting of the resistance value, R, in the circuit, and the dielectric loss and heat loss during the simulation. The effect of the bending on the antenna transmission characteristics can be explained from the perspective of circuit frequency characteristics, and the calculation time is significantly reduced.

## 4. Experiment and Comparison

**Figure 9:**
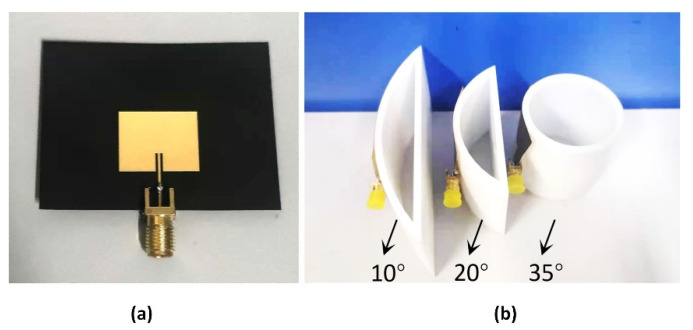
(**a**) Experimental sample of PI substrate antenna. (**b**) The cylindrical surface of the resin material manufactured based on 3D printing technology.

**Figure 10:**
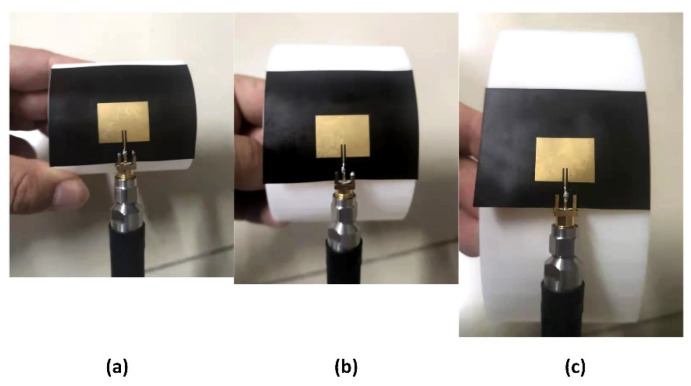
Using the vector network analyzer to test the S-parameters of the antenna experiment sample: (**a**) 35°, (**b**) 20°, and (**c**) 10°.

As shown in Figure 9a, the sample patch antenna was fabricated on a PI film with a thickness of 0.254 mm, which is a flexible, non-elastically deformable material. The back is the ground plane, sized 32 mm × 50 mm. In order to ensure accurate and stable bending angles, the photosensitive resin 3D printing technology was used to make cylindrical stents, as shown in Figure 9b. The patch antenna adopts side-feed mode to ensure that users can wear it comfortably. As shown in Figure 10, the S-parameters are measured for the flat state and three different E-plane bending states, with a vector network analyzer (R&S^®^ZNB20 Rohde&Schwarz, Munich, Germany).

**Figure 11:**
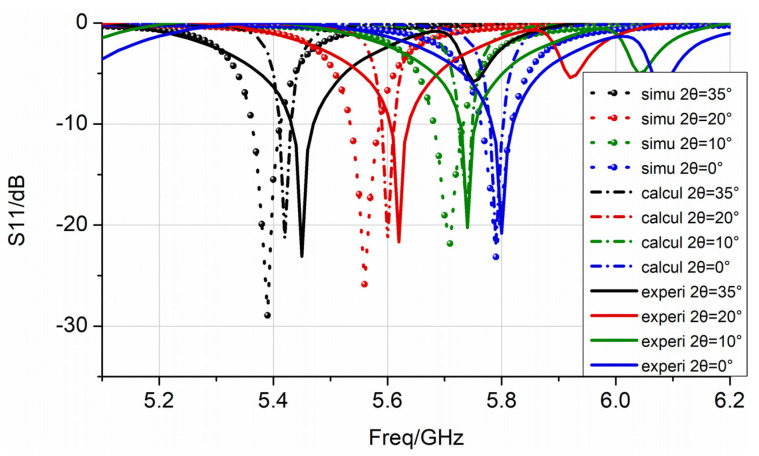
The full-wave simulation, theoretical calculation results, and experimental data, represented by dotted, dashed, and solid lines, respectively. The S-parameters for bending angles of 0°, 10°, 20°, and 35° are represented by blue, green, red, and black lines, respectively.

The results of nine samples under four bending states were measured. The maximum and minimum values of the resonance frequency in the results were removed, and the average value was calculated to reduce measurement errors. As expected from the simulation and theoretical calculations, the antenna’s resonant frequency decreased as the E-plane bending angle increased. The full-wave simulation, theoretical calculation results, and experimental data are shown in Figure 11.

It can be concluded from Figure 11 that, compared with the full-wave simulation and experimental results, the theoretical results are basically in accordance with both, as follows:

(1) At the resonance frequency, the maximum difference between the experimental and the simulation frequency was 1.11%, whereas it was only 0.55% between the experimental and theoretical calculations. This slight difference may be due to the deviation of the dielectric constant of the polyimide material from the calibration, or the size error of the patch during processing.
(2) For the amplitude at the resonant frequency point, the theoretical calculations were very close to the experimental data. The amplitude deviation of the simulation may be due to the position of the port and the feeding point, which affects the simulation result of the impedance. The S-parameter of the tested antenna exhibited a slight depression at high frequencies, which is caused by the excitation of higher resonance modes. The results show that the analysis method in this paper can reflect the bending effect of the patch antenna transmission.

## 5. Conclusions

This study analyzed the bending effect of the transmission characteristics of ultra-low-profile antennas suitable for wearable communication devices. For a patch antenna with E-plane bending angles of 0–35°, the effective dielectric constant method frequency prediction expression was derived. The effective dielectric constant was used to calculate the lumped element parameters. Impedance characteristics and the S-parameters of the bent antenna were constructed according to the frequency characteristics of the circuit. The measurement, simulation, and theoretical calculation results were very consistent. This direction lays the foundation for subsequent work: robust analysis of the transmission performance of antennas deformed by disturbances such as mechanical vibration, fast impedance matching of frequency reconfigurable antennas, and optimal design of passive structures to compensate for the bending effect.

## Figures and Tables

**Table 1:** Equivalent circuit lumped element parameters with different E-plane bending angles.

Circuit Parameters	E-Plane Bending Angles							
	0°	5°	10°	15°	20°	25°	30°	35°
$\varepsilon_{eff}$	3.68	3.73	3.75	3.88	3.93	4.04	4.09	4.2
$R_{\mathrm{calcu}}(\Omega )$	394.42	391.84	391.11	384.04	381.86	379.11	374.28	369.18
$C_{calcu}(pF)$	24.93	25.27	25.4	26.28	26.65	27.35	27.7	28.45
$f_{calcu}(GHz)$	5.79	5.75	5.73	5.64	5.6	5.53	5.49	5.42
